# Cut off value of technetium uptake in the differential diagnosis of Graves^,^ disease and subacute thyroiditis

**DOI:** 10.22038/aojnmb.2019.14241

**Published:** 2020

**Authors:** Demir Fadime

**Affiliations:** Department of Nuclear Medicine, Faculty of Medicine, Tokat Gaziosmanpasa University, Tokat, Turkey

**Keywords:** Technetium uptake, Graves, Subacute thyroiditis

## Abstract

**Objective(s):**

The aim of this study was to determine whether technetium (^99m^Tc) uptake is a relevant method for the differential diagnosis of Graves disease and subacute thyroiditis and calculate its cutoff value in case of its relevancy.

**Methods:**

A total of 69 patients, who were followed up (>3 months) in our hospital for thyrotoxicosis within 2015-2019 were enrolled in the study. Out of these 69 subjects, 39 patients had been diagnosed with Graves disease, and 30 of them had subacute thyroiditis. Biochemical parameters, thyroid scintigraphy, and ^99m^Tc uptake test results were evaluated.

**Results:**

^99m^Tc uptake was significantly higher in the patients with Graves disease than in the patients with subacute thyroiditis (P<0.001). Based on the ROC analysis the ^99m^Tc uptake cutoff value of 1.55% had an accuracy of 92.9%, with the sensitivity and specificity of 92% and 87%, respectively.

**Conclusion:**

In conclusion, the results of our study suggested that ^99m^Tc uptake test could be used in the differential diagnosis of Graves disease and subacute thyroiditis. The cutoff value of 1.55% for ^99m^Tc uptake test may guide in establishing a differential diagnosis between the two diseases.

## Introduction

 Thyrotoxicosis is a clinical condition characterized by hypermetabolism caused by the circulation of excessive thyroid hormones (thyroxine and/or triiodothyronine). This disorder may occur due to the excessive synthesis of hormones in the thyroid gland, excessive release of previously synthesized thyroid hormones, or excessive release of hormones from an endogenous or exogenous extrathyroidal source (1). 

 Thyroiditis (acute and subacute thyroiditis), which occurs due to the damage of the thyroid gland, is one of the most common causes of hyperthyroidism due to excessive hormone release. Graves and subacute thyroiditis (painless) are two diseases that are challenged in differential diagnosis in patients with thyrotoxicosis. 

 Radioactive iodine uptake (RAIU) test is used after the implementation of serum and serological tests to differentiate these two similar diseases (3). It is a quite useful method for this purpose. However, it needs measurment at 24-hour after administration of I-131, and the received radiation dose is relatively high. Therefore, it is not widely used. 

 The evaluation of the activity of thyroid tissue according to the activity in the salivary glands in thyroid scintigraphy provides a visual estimation of ^99m^Tc uptake. The ^99m^Tc uptake test, performed semiquantitatively using the region of interest (ROI) around the thyroid tissue and dose injector in thyroid scintigraphy, was offered as an adjunctive test in differential diagnosis (4). However, there are not enough studies in the literature examining the usefulness of thyroid scanning for this purpose. 

 The aim of this study was to determine whether

^99m^Tc uptake is a relevant method in the differential diagnosis of Graves disease and subacute thyroiditis and calculate a cutoff value for ^99m^Tc uptake test which is practical and easily applicable in the differential diagnosis of the two diseases.

## Methods

***Patients***

 This retrospective study included 69 patients who were admitted to our hospital due to thyrotoxicosis and received follow-up within 2015-2019. Blood biochemical parameters, thyroid scintigraphy, and ^99m^Tc uptake records, which were performed simultaneously, were evaluated retrospectively. 

^99m^ **Tc uptake test**

**Figure 1:**
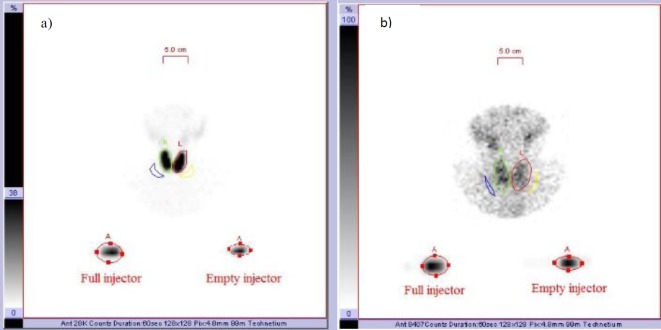
Calculation of thyroid uptake; a) calculation of uptake in a patient with Graves disease, b) calculation of uptake in a patient with subacute thyroiditis

 In oder to calculate ^99m^Tc uptake, SIEMENS Symbia gamma camera was used for imaging with a low-energy pinhole collimator. Images of the injector were obtained just before and after radiopharmaceutical injection. Thyroid gland imaging was performed 20 min after radiopharmaceutical injection at 5 mCi. The ^99m^Tc-pertechnetate thyroid uptake was calculated semi-quantitatively based on the ROIs in the thyroid tissue, background activity, and injector activity values using the following formula: 

Thyroid uptake (%)=Thyroid activity˗background activity/full injector-empty injector (Figure 1)

***Serological measurements***

 Thyroid stimulating hormone (TSH), fT_4_, and thyroid peroxidase antibody (anti TPO) levels were measured using the Dimension Clinical Chemistry System (Dade Behring Inc. Newark, DE), Roche Elecsys 2010, and Modular Analytics E170 (Elecsys module) immunoassay analyzers (Roche Diagnostics GmbH, D-68298, Mannheim, Germany) in one laboratory.

***Diagnosis of patients***

 The definitive diagnosis of the patients was made by an experienced endocrinologist based on thyroid scintigraphy, laboratory findings, and clinical data in the follow-up. Graves disease was managed with clinical and successful treatment with antithyroid drug, surgery, or radioactive ablation. Subacute thyroiditis was defined by the spontaneous resolution of symptoms with the normalization of thyroid function tests at follow-up.

***Statistical analysis***

 Continuous variables were reported as mean ±standard deviation or median values and ranges. Categorical variables were reported as absolute numbers. The differences between the groups were evaluated by Student's t-test, Mann-Witney U test, and Chi-square test. A cutoff value was calculated by ROC analysis using uptake values to distinguish Graves patients from subacute thyroiditis patients. Values less than 0.05 were considered significant. All analyses were performed in SPSS software (version 20.0).

## Results

 A total of 69 patients (45 female and 24 male) were included in the study. In this regard, 39 patients were diagnosed with Graves disease, and 30 patients had subacute thyroiditis. The mean age was 49.07±17.03 years. There was a significant difference between the two groups of patients in terms of ^99m^Tc uptake, TSH, fT4, and anti-TPO antibodies. However, there was no significant difference between them in terms of age and gender (Table 1).

**Table 1:** Comparison of parameters between the studied groups

	**Graves disease** **(n=39)**	**Subacute thyroiditis** **(n=30)**	**P-Value**
**Age** Mean±SD (range)	46.49±16.29 (19-74)	52.43±18.27 (22-84)	0.159
**Gender** (F/M)	25/14	20/10	0.515
**TSH (µIU/mL)** Mean±SD (range)	0.029±0.089 (0.005-0.544)	0.085±0.143 (0.005-0.718)	**<0.001**
**fT**_4_ ** (ng/dL)** Mean±SD (range)	3.283±1.909 (1.01-7.77)	2.307±1.257 (0.00-4.89)	**0.031**
**Anti-TPO(IU/mL)** Mean±SD (range)	169.1±180.42 (5-600)	57.31 ±110.197 (9-499)	**0.011**
**Anti TPO -/+**	13/25	21/7	**<0.001**
^99m^ **Tc uptake** Mean±SD (range)	5.803±5.778 (0.2-26.6)	0.730 ±0.993 (0.0-3.9)	**<0.001**

Bolded p-values indicate statistical significance at P<0.05. TSH: thyrotropin, fT_4_: free thyroxine, anti-TPO: anti-thyroid peroxidase antibody, ^99m^Tc uptake: technetium uptake

 The TSH level was significantly lower in the Graves group than in the subacute thyroiditis group. On the other hand, the fT4 level, anti-TPO level, and anti-TPO positivity were higher in the Graves group (Table 1). Comparison of ^99m^Tc uptake between the two groups revealed a 

significantly higher value in the Graves group (P<0.001).

**Figure 2:**
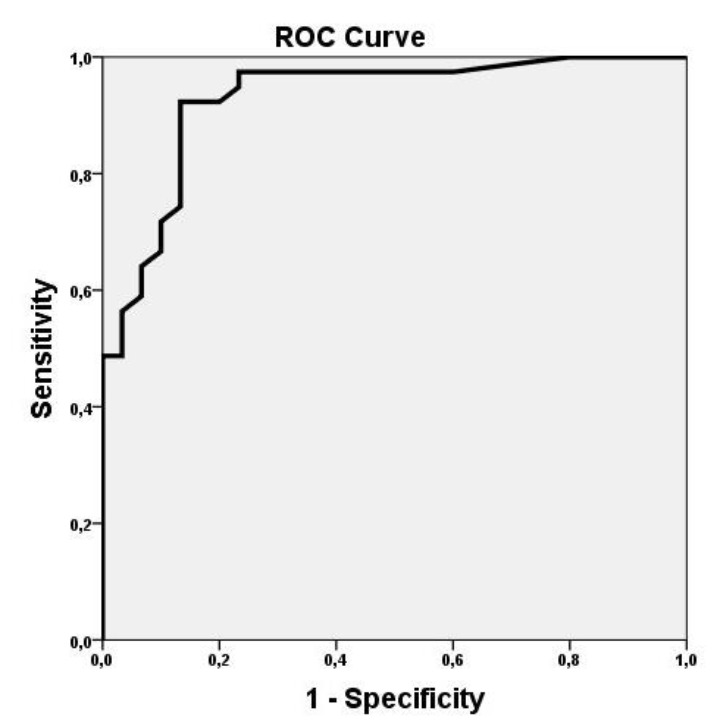
Receiver operating characteristic curve for ^99m^Tc uptake in the differential diagnosis of Graves disease and subacute tyroiditis

 Based on the results of ROC analysis, the accuracy for the cutoff value of 1.55% was obtained as 92.9% with a sensitivity and specificity of 92% and 87%, respectively (Figure 2).

## Discussion

 The RAI uptake test is a diagnostic method recommended in the guidelines for patients with thyrotoxicosis that could not be given a differential diagnosis based on clinical and biochemical tests (5). However, the implementation of the test is time-consuming and laborious. Thyroid scintigraphy is one of the most frequently used tests in the evaluation of thyrotoxicosis. One of the first studies on the calculation of ^99m^Tc uptake by semi-quantitative evaluation was a study conducted by Maisey et al. They reported that ^99m^Tc uptake test is a fast and cost-efficient method which can be used as an alternative to RAI uptake (4). 

 The results of a study performed in the same period showed that ^99m^Tc uptake and RAI uptake tests correlated well (6). Thereafter, nfew studies were performed in this field. Eventually, in recent years, with the widespread use of automatic ^99m^Tc uptake programs, this test has begun to be a routine measure; accordingly, studies have been published on the normal range of values for ^99m^Tc uptake (7, 8). 

 In a study conducted by Mccauley et al., the normal value of ^99m^Tc uptake for the UK community was in the range of 0.2-2.0% (7). In addition, ^99m^Tc uptake test has been also suggested for both diagnosis and detection of the recurrence of Graves disease in recent studies (9, 10). Singhal et al. found a strong relationship between Graves recurrence and ^99m^Tc uptake value (9). 

 Baskaran et al. showed that in the pediatric patient group, ^99m^Tc uptake had high sensitivity and specificity in the differential diagnosis of Graves diseases and diseases characterized by the excessive release of thyroid hormones (10). Therefore, they concluded that the test can be used for differential diagnosis, especially in patients who cannot be definitively distinguished with serology. 

 In our study, we investigated whether the ^99m^Tc uptake test can be a relevant test for the differential diagnosis of Graves disease and subacute thyroiditis in thyrotoxicosis. According to our results, ^99m^Tc uptake values were significantly higher in the Graves group than in the subacute thyroiditis group. In addition, when we performed the ROC analysis, the cutoff value of the ^99m^Tc uptake test for the differentiation of the two diseases was obtained as 1.55%, which rendered very high accuracy, sensitivity, and specificity (92.9%, 92%, and 87%, respectively). A similar study was conducted by Uchida et al. (11). They reported a cutoff value of 1% with the sensitivity and specificity of 96.6 and 97.1, respectively. The results of the mentioned study were similar to the our findings.

## Conclusion

 Our results suggested that the consideration of a cutoff value of 1.55% for ^99m^Tc uptake might make it a proper supplemental test in the differential diagnosis of Graves disease and subacute thyroiditis in patients with thyrotoxicosis.

## Conflicts of interest

 The authors declare no conflicts of interest.
